# Genome-Wide Identification and Expression Profiling of the Invertase Genes Involved in Sugar Metabolism and Accumulation in *Actinidia arguta*

**DOI:** 10.3390/ijms26052150

**Published:** 2025-02-27

**Authors:** Xu Qiang, Ting Ren, Ying Zhang, Yun Jia

**Affiliations:** 1 Xi’an Botanical Garden of Shaanxi Province (Institute of Botany of Shaanxi Province), Xi’an 710061, China; 2 Key Laboratory of Resource Biology and Biotechnology in Western China (Ministry of Education), College of Life Sciences, Northwest University, Xi’an 710069, China

**Keywords:** *Actinidia arguta*, expression profile, kiwifruit, Invertase, sugar metabolism, transcription factors

## Abstract

Invertase (INV, EC3.2.1.26) is widely recognized as an indispensable enzyme for catalyzing sucrose degradation and plays a central role in plant growth as well as fruit quality improvement. However, no systematic study has been performed in kiwifruit. Here, we identified 102 *AaINV* genes in the *Actinidia arguta* “M1” genome. Their physical and chemical properties, subcellular localizations, phylogenetic relationships and expression profiles were characterized. Phylogenetic analysis showed that the *INV* members were clustered into three groups (vacuole invertases (VINVs) and cell wall invertases (CWINVs) in Group I, alkaline/neutral invertase (NINVs) in Group II and Group III), demonstrating evolutionary conservation in the INV family across *Arabidopsis* and *Actinidia* species. Gene replication analysis revealed that many *AaINV* genes were derived from gene duplication events. Molecular evolution analysis based on Ka/Ks ratios indicated that the *INV* members have experienced extensive purifying selection during evolution. To explore the potential gene functions, we integrated RNA-seq and metabolomics to analyze *AaINV* gene expression patterns and sugar accumulation in three *A. arguta* varieties (“Kukuwa”, “Qinhuang”, “Xianziguang”), respectively. The expression analysis of the 102 genes showed that the expression patterns varied among the three kiwifruit varieties at fruit maturity stage. The expression levels of *AaINVs* were also investigated via qRT-PCR in these varieties. Specifically, we constructed a complex regulatory network that regulates sugar metabolism in kiwifruit based on the correlation between 42 *AaINV* genes and 14 sugar metabolites. These findings provide insights into physiological functions of *AaINVs* in kiwifruit, especially roles in governing sugars accumulation in fruits.

## 1. Introduction

Sucrose (Suc) is the major transport form of the photosynthetic carbohydrate through the phloem to sink tissues (e.g., fruits, roots and shoot tips) and plays central physiological roles in plants [1,2]. The transported sucrose in different cells is hydrolyzed to hexose through sucrose synthase (SUS, EC2.4.1.13) or sucrose convertase (INV, EC3.2.1.26) to provide carbon and energy sources for numerous metabolic pathways [3]. INVs (also known as β-fructosidases) catalyze the cleavage of sucrose into fructose and glucose, which are present in both photosynthetic and non-photosynthetic tissues [4]. The function of INVs is available for multiple biological processes, such as carbohydrate partitioning, organ building, response to biotic and abiotic stresses, etc. [4,5]. According to the pH values, *INVs* can be referred as acid invertases (AINVs, pH 4.5–5.0) and neutral/alkaline invertases (NINVs, pH 6.5–8.0). Moreover, it can also be assigned into three categories based on their subcellular compartments. NINVs are usually located in chloroplasts, mitochondrion or cytoplasm, whereas AINVs appear in the cell wall or vacuole, corresponding to CWINVs and VINVs, respectively [6,7]. Despite being localized in different cellular compartments, CWINVs and VINVs share similar biochemical properties [8]. Both enzymes catalyze the hydrolysis of sucrose and other β-fructose-containing compounds, such as oligosaccharides, which is why they are also referred to as β-fructofuranosidases [9]. In contrast, NINVs lack an N-terminal signal peptide and do not function as β-fructofuranosidases; instead, they specifically catalyze sucrose hydrolysis [10].

Up to date, the INV members have been extensively documented in diverse plants, such as *Arabidopsis* [11,12], rice [13], tobacco [4,14] and tomato [15,16]. The functional characterization of INVs and their regulatory mechanisms has also been extensively studied and documented. It is widely believed that CWINVs are crucial for various physiological and developmental processes in plants, including sucrose partitioning [17], seed and pollen development [18] and environmental responses [19,20]. The evidence also indicates that VINVs are involved in the functioning of fruits and storage organs [21], as well as in plant responses to drought and hypoxia stress [22]. The proposed functions of NINVs have also been increasingly recognized, encompassing sugar signal transduction [23], shoot and root growth [24], fruit development [25,26,27,28] and responses to both biological and abiotic stresses [20,29]. For instance, rapid glucose and fructose accumulation in the leaves of *MeNINV1*-overexpressing *Arabidopsis* is attributed to higher A/N-INV activity [30]. In apple (*Malus domestica*), the expression of two *INV* genes, *CWINV* and *NINV*, is significantly downregulated, accompanied by an decrease in sugar content during fruit development [25]. In tobacco (*Nicotiana tabacum*), inhibition of the expression of *NtNINV10* lowers the levels of glucose and fructose in leaves, while *NtNINV10* is induced by drought and salinity stresses [4]. Moreover, some transcription factors (TFs), e.g., *NAC71*, *WRKY3* and *WRKY57*, have important regulatory implications for sugar accumulation mainly by activating the expression of *INV*, *SPS* and *SUS* [26]. Consequently, the *INV* family members involved in sucrose metabolism and the associated TFs are essential for understanding the regulatory mechanisms of sugar accumulation in plants [27,28]. However, the *INV* gene family has not yet been thoroughly investigated at the whole-genome level in fruit crops.

Kiwifruit (*Actinidia* spp.) is an economically significant fruit crop with exceptional nutritional benefits and remarkable health-promoting properties [31]. In recent years, consumers are attracted to its unique fruits, fresh colors and high nutrition, especially those of *A. arguta*, which has a smooth, hairless skin, green or purple color and contains high levels of anthocyanin [32,33]. Although *A. arguta* is known as a wild species, it is also commercially cultivated worldwide and introduced to the market due to its special desirable traits [34,35]. In *A. arguta*, the nutrient composition, functional activity and flavor quality vary considerably among cultivars [36,37,38]. Previous studies have identified that sucrose is the predominant sugar in *A. arguta*, followed by glucose and fructose [39,40]. However, there is still limited understanding of the relationship between fruit sucrose accumulation and *INV* genes. Hence, it is essential to characterize the *INV* gene family in kiwifruit to investigate its potential role in sugar accumulation.

Recently, the kiwifruit genome (*A. arguta* “M1”) has been sequenced [41], which laid a solid foundation for genome-wide analysis of *INV* genes in kiwifruit. In this study, we focused on genome-wide identification, phylogenetic analysis, gene structure, collinearity analysis and cis-acting elements of 102 *INV* genes in *A. arguta*. The transcriptome data were employed to assess the relative expression of *AaINV* genes in three *A. arguta* varieties, namely “Kukuwa” (KKW, full-green flesh), “Qinhuang” (QH, yellow) and “Xianziguang” (XZG, purple). In addition, several metabolites accumulated in the fruit samples were screened as hub players in flesh sweetness during fruit quality formation. We further analyzed their correlation of *AaINVs*, TFs and sugars to construct the regulation network. This comprehensive analysis provides valuable insights into the functional roles of the *INV* genes in fruit crops.

## 2. Results

### 2.1. Genome-Wide Identification of INVs in A. arguta

In the present study, a total of 102 non-redundant AaINVs belonging to NINVs (72), CWINVs (23) and VINVs (7) were identified in the kiwifruit (*A. arguta* “M1”) genome, and these were labelled AaNINV1 to AaNINV72, AaCWINV1 to AaCWINV23 and AaVINV1 to AaVINV7, respectively (Table S2). Nearly 78% of AaINVs had a length of about 500 amino acids. Notably, AaNINV25 encoded the shortest protein (81 amino acids), whereas AaVINV7/AaCWINV23 had an exceptionally longer size of 1187/1076 amino acids. The theoretical isoelectric point (pI) values of the AaINV genes varied from 4.64 (AaCWINV12) to 9.76 (AaNINV2), and the molecular weight ranged between 8.95 kDa (AaNINV25) and 132.27 kDa (AaVINV7), with an average range of 61.93 kDa (Table S2). The subcellular localization of the most of AaNINVs (62/72 = 86.11%) was in the plasma membrane, followed by the cytoplasm (21/72 = 29.17%) and chloroplast (18/72 = 25%). By contrast, the majority of AaCWINVs and AaVINVs were localized in the vacuole, and the others were in the lysosomal and extracellular matrix (Figure S1).

### 2.2. Phylogenetic Tree Construction

**Figure 1:**
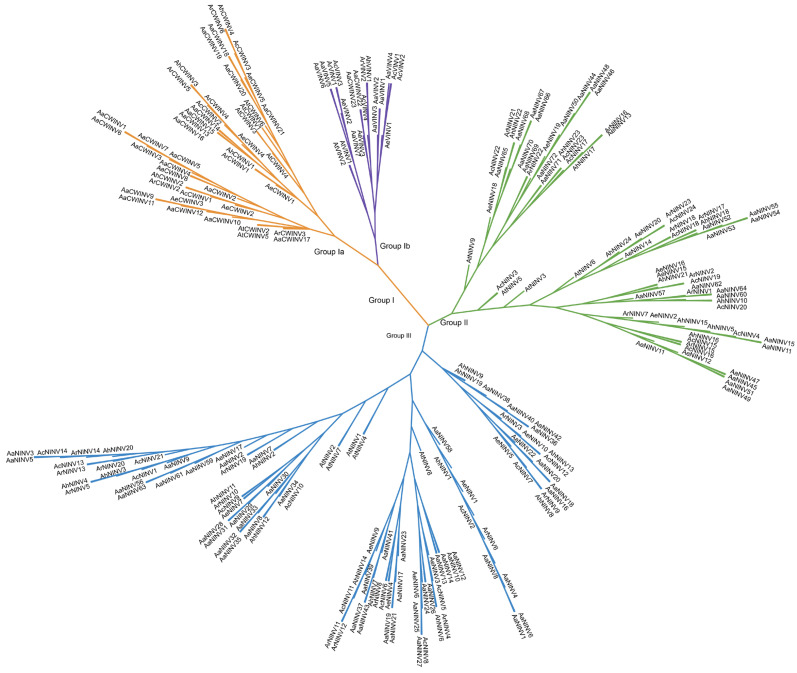
Phylogenetic analyses of the INV families from five *Actinidia* species and *Arabidopsis*. Clades with different colors represent diverse subgroups. Species abbreviations are listed as follows: At: *A. thaliana*; Aa: *A. arguta*; Ac: *A. chinensis*, Ae: *A. eriantha*; Ah: *A. hemsleyana*; Ar: *A. rufa*.

To further investigate the evolutionary relationships of INV proteins, an NJ phylogenetic analysis containing 17 protein sequences from *Arabidopsis* and 221 from *A. arguta*, *A. chinensis*, *A. eriantha*, *A. hemsleyana* and *A. rufa* was performed (Tables S2 and S3). The INV gene family from the six species was classified into three main phylogenetic clades (Group I, Group II and Group III), in which Group III and Group I were the largest and smallest subgroup with 101 and 66 INV proteins, respectively (Figure 1). *NINVs* could be subdivided into Group II and Group III. Group I further divided into two distinct subgroups, Group Ia and Group Ib, which correspond to the VINVs and CWINVs located in the vacuole and cell wall, respectively (Figure 1). The results above indicated that INV proteins of five *Actinidia* species and *Arabidopsis* were unevenly distributed in all subgroups. The phylogenetic analyses suggest that the INVs in each branch evolved independently.

### 2.3. Gene Structure and Conserved Motif of AaINVs

**Figure 2:**
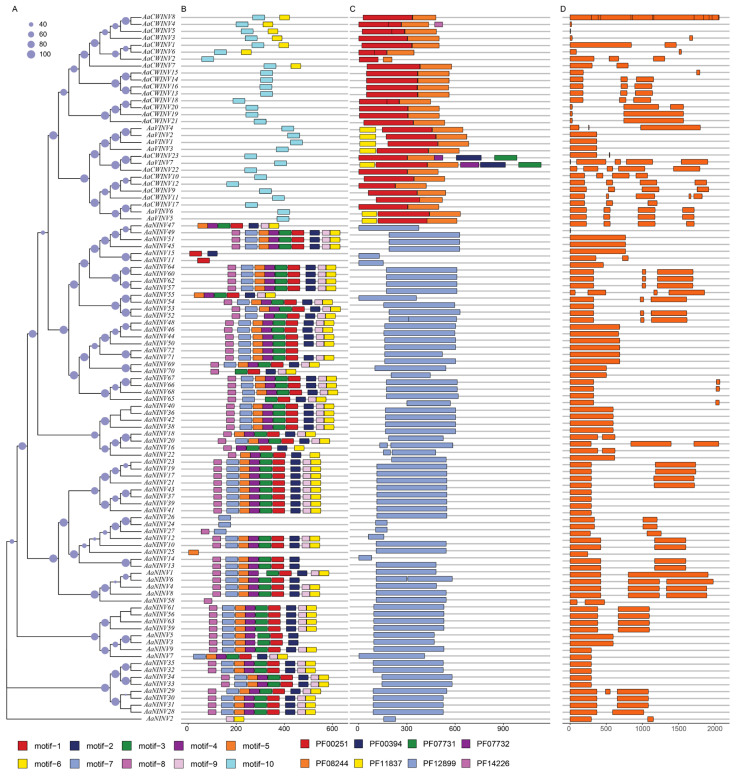
(**A**) Neighbor-joining trees constructed for AaINVs. (**B**) Protein motifs. The number of the motifs 1–10 are presented in different colored boxes. (**C**) Conserved domains. Different domains are displayed by colored boxes. (**D**) The gene structure of *AaINVs*, with orange boxes representing CDS.

To examine the characteristic regions of AaINV proteins, the conserved motifs for each AaINV were explored by MEME v5.5.3. There were a total of 10 motifs identified in the AaINVs, and more than 50% of AaNINV members had nine motifs, corresponding to a specific domain PF12899 (Figure 2). However, motif 10 were exclusively found in the subgroups AaCWINV and AaVINV and were located on two functional domains (PF00251 and PF08244), but could not be presented in AaNINV, indicating a functional divergence within the AaINV gene family. We further compared the DNA sequences and examined the intron/exon arrangement of each *AaINV* gene in kiwifruit. In total, the number of exons in the *AaINVs* ranged from one to thirteen, and genes from the same category displayed high similarity. Notably, the *AaVINVs* possessed the most exons, with an average of only 1.7, whereas *AaCWINVs* typically contained three or more exons on average (Figure 2).

### 2.4. Chromosomal Distribution and Gene Duplication Analyses of the AaINV Gene Family

**Figure 3:**
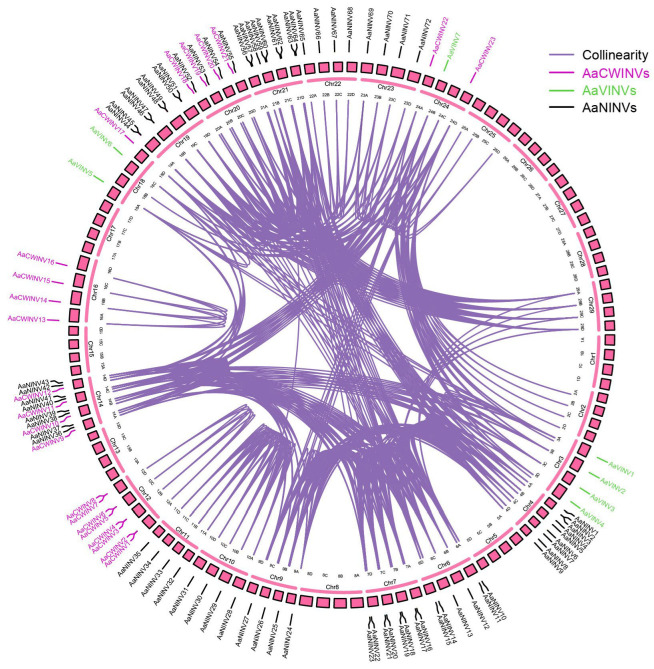
Chromosome distribution and collinearity analysis of *AaINVs* in the kiwifruit genome. The magenta, green, and black fonts represent the *AaCWINV*, *AaVINV* and *AaNINV* genes, respectively. The purple lines represent collinear *AaINV* gene pairs, and the pink boxes present the chromosome.

According to the genome annotation of *A. arguta*, the 102 identified *AaINV* genes were unevenly distributed on 17 of the 29 chromosomes (Chrs). The largest number of *AaINVs* was mapped onto Chr14 with 12 *AaNINV* and 4 *AaCWINV* genes, followed by Chr4/21 with 9 *AaNINV* members each. Chr7/12/19/20 contained eight *AaINVs* members each, and only two genes, *AaCWINV17* and *AaVINV5*, were located on Chr18 (Figure 3). Notably, we observed that some genes within the same subfamily displayed gene clusters, such as the *AaNINV* cluster on Chr4, Chr19 and Chr21, *AaCWINV* on Chr12 and *AaVINV* on Chr3 (Figure 3).

To further explore the expansion pattern of the *AaINVs*, we detected three types of duplication events throughout the *AaINV* genes. In total, 97 out of 102 *AaINVs* (95.10%) experienced whole-genome duplication (WGD) events in kiwifruit according to the MCScanx method (Table S4). Otherwise, only *AaCWINV4* underwent tandem duplication (TD), and three *AaCWINV* genes (*AaCWINV2*, *AaCWINV6* and *AaCWINV8*) exhibited proximal duplication (PD) (Figure 3; Table S4). The duplication analysis indicated that WGD events were the driving force for the expansion of *INVs* in kiwifruit.

### 2.5. Collinearity Analysis and Selective Pressure

**Figure 4:**
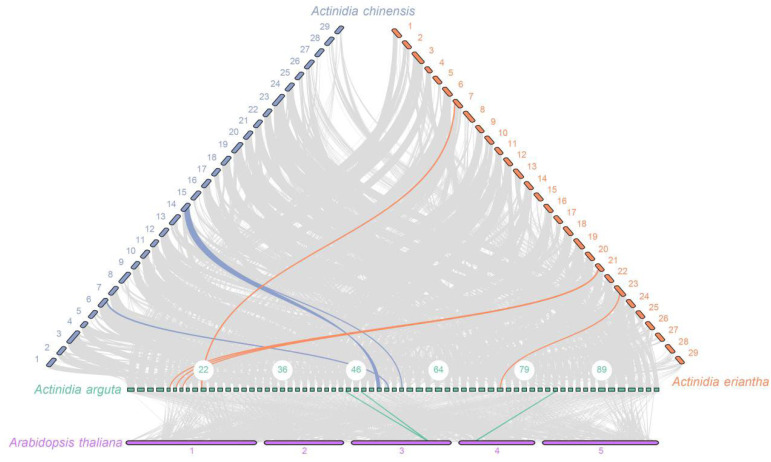
Synteny analyses for *INV* gene family between *A. argute* and *A. thaliana*, *A. chinensis* and *A. eriantha*, respectively. The gray lines in the background show the collinear blocks within *A. arguta* and other plant genomes; the colored lines indicate orthologous gene pairs.

To investigate the potential evolutionary clues of *INVs*, a comparative syntenic map was constructed, comparing *A. arguta* with three other species (*Arabidopsis*, *A. chinensis* and *A. eriantha*). The collinearity predictions presented three *INV* orthologous gene pairs shared between *Arabidopsis* and *A. arguta*. Meanwhile, we observed that there were three and five pairs of collinear *INV* genes shared with *A. chinensis* and *A. eriantha*, respectively (Figure 4). We also noticed that the collinear genes clustered within the same evolutionary branch (Figure 2), suggesting that these genes may share similar functions.

Furthermore, Ka/Ks ratios were computed to understand evolutionary selection for the duplicated *AaINV* genes (Table S5). We found that the majority of Ka/Ks ratios for *AaINV* gene pairs were <1 (Table S5), indicating that these genes experienced extensive purifying selection during evolution. Interestingly, positive selection (Ka/Ks > 1) occurred in the *AaCWINV* gene pair (*AaCWINV18*/*AaCWINV21*), suggesting that the *AaCWINV* genes may have undergone positive selection and experienced a rapid evolutionary rate during kiwifruit domestication.

### 2.6. Cis-Acting Element Analysis of AaINV Gene Family

**Figure 5:**
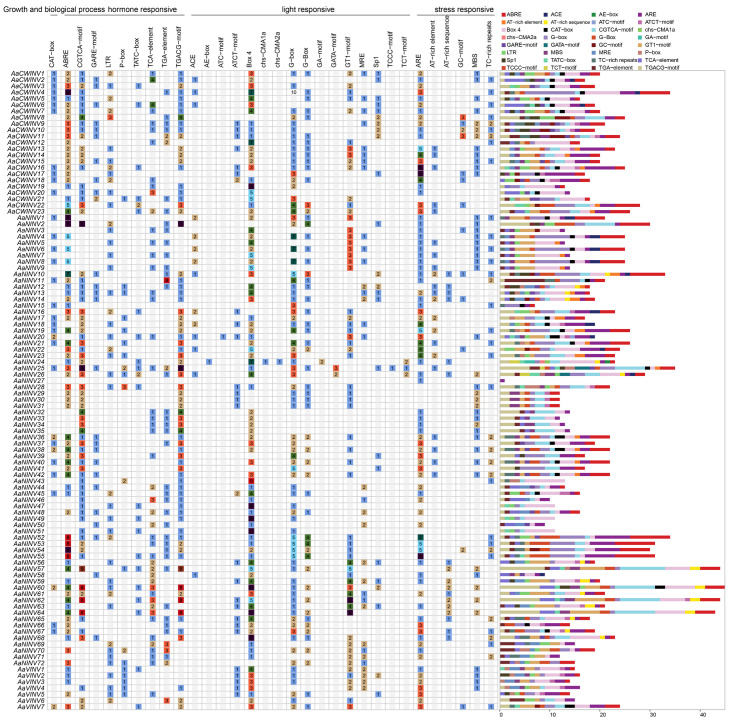
Cis-elements prediction in the 2000 bp region upstream from the start codon of *AaINVs*. The distribution of these cis-elements is illustrated on the right, with different colors showing diverse types of cis-elements. The number of cis-elements is presented in the colored box.

The identified cis-elements were assigned to the following four distinct groups: growth and biological process, hormone-responsive, light-responsive and stress-responsive, based on their putative functions. Among them, several common cis-elements that were widely observed in the promoter regions of most *AaINVs*, including four responsive to methyl jasmonate (MeJA, CGTCA-motif and TGACG-motif), abscisic acid (ABA, ABRE) and salicylic acid (SA, TCA-element), two responsive to light (Box 4 and G-box) and two responsive to abiotic stress (ARE and MBS) (Figure 5). Interestingly, all *AaINV* members possessed the cis-elements associated with photoresponsiveness, which suggests that *AaINVs* are broadly involved in the transport of synthesized photoglycosides and other secondary metabolites. Additionally, we found that varying numbers of regulatory elements were identified in the upstream promoter regions of the *AaINVs*. For instance, 13 to 34 regulatory elements occurred in the promoter region of the *AaCWINVs*, while 14 to 24 were identified in *AaNINVs* (Figure 5). The diversity of cis-acting elements in the promoter regions of *AaINVs* suggests that their functions may be regulated by a complex network involving stress responses, hormonal regulation and plant growth and development.

### 2.7. Genome-Wide Expression Analysis of AaINV Genes in Different Varieties

**Figure 6:**
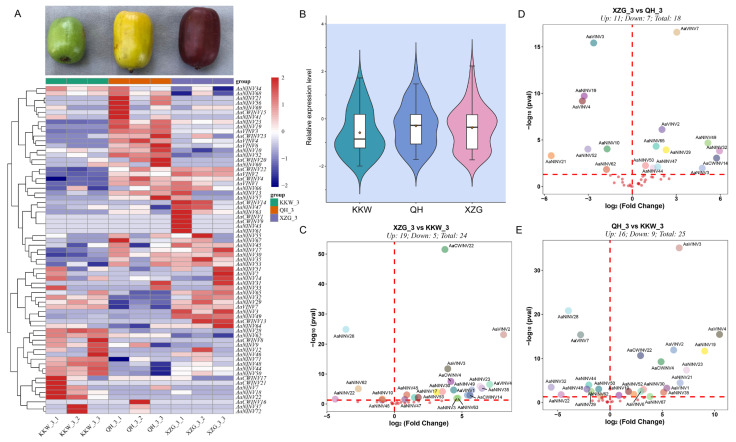
(**A**) Color observation and heatmap of the expression from three kiwifruit varieties. KKW, QH and XZG represent the green-, yellow- and purple-colored flesh samples, respectively. (**B**) Expression profiles of 102 *AaINV* genes in the three varieties. (**C**–**E**) Volcano plot from different varieties between XZG vs. KKW, XZG vs. QH and QH vs. KKW, respectively.

To further explore the functions of the *AaINV* family genes, we examined their expression patterns in three *A. arguta* varieties using RNA-seq data (Table S6). A total of 102 transcripts were annotated as *AaINVs* in *A. arguta*, as shown in Figure 6. Pearson correlation coefficient analysis indicated that their expression levels were highly correlated (Pearson’s r > 0.85, *p* < 0.05), such as *AaCWINV1*/*AaCWINV9*, *AaNINV32*/*AaNINV65*, *AaCWINV4*/*AaVINV6* and *AaVINV7*/*AaNINV29*. However, the expression patterns of *AaINV* genes varied at fruit maturity stage among different *A. arguta* varieties (Figure 6A,B and Figure S2). Specifically, we observed significant upregulation of 11, 19 and 16 *AaINV* genes, respectively, and downregulation of 7, 5 and 9 genes, respectively, between XZG vs. QH, XZG vs. KKW and QH vs. KKW (Figure 6C,D). For instance, *AaVINV3*, *AaVINV4*, *AaVINV7* and *AaNINV19* were significantly upregulated between XZG vs. QH and QH vs. KKW, whereas *AaCWINV22*, *AaNINV28*, *AaVINV2* and *AaVINV3* were specifically upregulated between XZG vs. KKW. Interestingly, several *AaINV* genes exhibited significantly differential expression among varieties, e.g., *AaVINV3*, *AaVINV4*, *AaNINV19* and *AaNINV21* (Figure S3). Notably, some *AaINV* genes displayed opposing expression patterns among different varieties. For example, *AaCWINV23* and *AaVINV6* were predominantly upregulated in QH but downregulated in XZG and KKW (Figure 6). These findings offer valuable insights into the diverse roles and regulatory mechanisms of *AaINVs*, highlighting their functional diversity and ubiquitous expression patterns.

### 2.8. GO Functional Annotation of AaINV Genes

To better understand the functional roles of *AaINV* genes, we used the GO database to analyze their potential biological roles. There were nine functional terms obtained with 102 *AaINVs* (Figure S4). The most abundantly enriched category was the sucrose catabolic process, endo-alpha-N-acetylgalactosaminidase activity and sucrose alpha-glucosidase activity, which are closely associated with the sucrose metabolic process. Secondly, nine *AaINVs* were enriched in the trehalose biosynthetic process, indicating that these genes play a crucial role in trehalose accumulation during the ripening of kiwifruit. Furthermore, the regulation of seed germination was also significantly enriched with four *AaINVs*, therefore *INVs* may potentially have key regulation roles during seed and fruit development.

### 2.9. Correlation Analysis Between AaINVs Expression and Sugar Content in Kiwifruit

**Figure 7:**
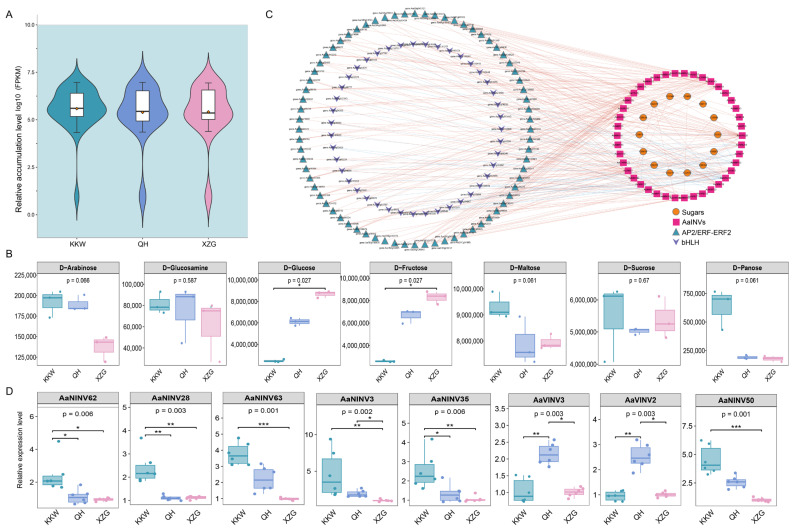
(**A**) Overview of the sugar metabolome profiles in three kiwifruit varieties. (**B**) Comparison of the seven representative sugar metabolites between three kiwifruit varieties. (**C**) The regulatory network of key sugar metabolites in kiwifruit. The orange circles represent sugars, and the rose red squares represent *AaINV* genes. Green triangles and blue arrows indicate AP2/ERF-ERF2 and bHLH transcription factors, respectively. (**D**) Results of qRT-PCR analysis of the five selected *AaINVs* in KKW, QH and XZG; * *p* < 0.05, ** *p* < 0.01, *** *p* < 0.001.

To analyze whether the expression levels of *AaINVs* are correlated with sugar metabolism measured in mature kiwifruit, we calculated the Pearson correlation coefficient (PCCs) between 102 *AaINVs* and 30 metabolites such as sucrose, fructose, glucose, etc. (Tables S7 and S8). Globally, the green samples (KKW) contained more sugar metabolites than the yellow and purple samples did (Figure 7A). In contrast, XZG samples had high fructose and glucose content as compared to that of KKW and QH; QH samples were more endowed with glucosamine metabolites (Figure 7B). These results suggest a high variation in the sugar metabolism of these three varieties.

A total of 42 *AaINV* genes, including 3 *AaCWINVs*, 6 *AaVINVs* and 33 *AaNINVs*, were screened according to GO enrichment, and their expression was highly correlated with the accumulation of various metabolites (Figure S5; Table S8). For instance, the expression patterns of *AaCWINV22* and *AaNINV18* were highly correlated with fructose, glucose and gluconic acid. Furthermore, the expression of three *AaINV* genes (*AaNINV3*, *AaNINV35* and *AaNINV63*) were significantly positively correlated with fructose and glucose content. However, *AaNINV18*, *AaNINV22*, *AaNINV28* and *AaNINV62* had a negative correlation with fructose and glucose concentration. In particular, the expression of *AaNINV12* was consistent with that of sucrose content, while *AaNINV13* and *AaNINV66* were negatively correlated with sucrose content.

To investigate the key TFs modulating the activation of the candidates encoding enzymes of sugar accumulation in kiwifruit, we further identified two TF families containing 176 bHLH and 222 AP2/ERF-ERF2. These candidates displayed expression specificity in the three kiwifruit varieties (Figure S6). Subsequently, we performed correlation analysis on the obtained candidates and 14 sugar components (Table S8). Furthermore, the potential regulatory networks mediated by these candidates were then constructed using Cytoscape v3.7.1 (PCC > 0.9, *p* < 0.05) (Figure 7C). Our analysis revealed strong connectivity between the TFs and the *AaINV* genes (e.g., *AaNINV28*, *AaNINV30*, *AaNINV49*, *AaVINV3* and *AaVINV6*), highlighting their role in regulating sugar metabolism during the ripening of kiwifruit.

### 2.10. Validation of AaINV Expression by RT-qPCR

To verify the results of RNA-seq, we checked the expression of five genes, including *AaVINV2*, *AaVINV3*, *AaVINV7*, *AaNINV45* and *AaNINV50* genes, via qRT-PCR in the three kiwifruit varieties (Figure 7D). The results indicated that the expression levels of these genes were altered in colored accessions. Notably, the expression levels of *AaVINV2*, *AaVINV3* and *AaVINV7* were significantly upregulated in QH, whereas the expression of *AaNINV45* and *AaNINV50* exhibited upregulation in KKW. These results suggest that *AaINV* genes play complex regulatory roles in kiwifruit.

## 3. Discussion

*INVs* have a pivotal role in coordinating carbohydrate metabolism, stress responses and sugar signaling, as well as improving fruit quality [5,42,43,44]. The first *INVI* was isolated in potato (*Solanum tuberosum*) [45] and then characterized as a large protein family in multiple plants, with 21 members in maize (*Zea mays*) [46], 29 in moso bamboo [7], 24 in tomato [16] and 36 in tobacco [4]. However, a genome-wide identification of the *INV* gene family in kiwifruit has not been reported. With the advancement of third-generation sequencing technology, a high-quality reference genome of *A. arguta* “M1” has been assembled [41], providing a valuable resource for analyzing the *INV* gene family in kiwifruit. In this study, we identified and systematically named 102 INV members based on their chromosomal positions in *A. arguta*, which exceeds the number of genes found in *Arabidopsis* and other *Actinidia* species (Table S3). This difference may have occurred because *INVs* have expanded in the *Actinidia* species. Gene duplication and divergence are known to play key roles in the expansion of gene families and the development of novel gene functions during evolution [47]. The kiwifruit genome has experienced at least three rounds of WGD, followed by segmental duplication and tandem duplications [41]. These events are believed to be responsible for the expansion of the *INV* gene family during evolution [7,48]. Similar results have also been discovered in bamboo and tobacco [4,7]. Nevertheless, most of the duplicated gene pairs showed a *Ka*/*Ks* ratio of less than 1, suggesting that the *ArINV* genes were subject to purifying selection, resulting in limited functional divergence after duplication.

Among the known classifications, the *INV* family is classified into two categories based on their cellular location: AINVs (including CWINVs and VINVs) and NINVs [6,16]. Our analysis demonstrated a high correlation between the phylogenetic relationships, intron–exon structures and protein motif distributions of *AaINV* members. The phylogenetic analysis clustered ArINVs into Ia and Ib, which correspond to the CWINVs and VINVs subgroups, respectively. The protein sequence analysis showed that two subgroups possessed different functional domains (PF11837 in the VINVs), which may account for the differences in their enzyme activities [49]. The NINVs were separated into group II and group III branches based on 169 NINVs from six species (Figure 1), aligning with the evolutionary characteristics of INVs [7,22]. The diversity observed in ArINVs suggests that they may have experienced distinct evolutionary pressures in kiwifruit.

*INV* genes play multiple functional roles in different fruit ripening, as is evident from their expression patterns in other plant species [25,28,42]. For instance, the overexpression of the *MiINV* genes demonstrates that during the mango ripening stage, the levels of sucrose and mannitol show an increasing trend [28]. In tomato, RNAi-mediated knockdown of the apoplastic *SlINVINH1* expression results in enhanced seed filling and higher sugar content in the fruits [42]. Here, the RNA-seq analysis revealed that the expression of *AaINVs* in XZG and QH fruits was higher than that in KKW (Figure 6). Combined with metabolome data, we observed in XZG and QH fruits that fructose and glucose increased and sucrose decreased, which was in line with the trend of expression of *AaINVs*. The complex and diverse expression patterns of *AaINVs* were further observed across three kiwifruit varieties, suggesting that they had different functions. For example, *AaNINV3*, *AaNINV35* and *AaNINV63* were highly expressed in XZG and showed a significant positive correlation with fructose and glucose content (Figure 6A and Figure S5). This finding aligns with studies on the homologous *NINV* gene in apple, which is known to play a crucial role in sugar metabolism and accumulation during fruit ripening [25]. The expression levels of *AaNINV18*, *AaNINV22*, *AaNINV28* and *AaNINV62* were higher in KKW compared to those in the other varieties and showed a negative correlation with fructose and glucose concentration (Figure 6A and Figure S5), suggesting their potential involvement in sucrose metabolism [5,6,25]. In contrast, the expression of some *AaINVs* was not detected in any of the varieties, suggesting that these may be pseudogenes, functionally redundant, or possibly expressed in other specific tissues or under certain conditions [50,51]. In addition, we selected eight *AaINV* members that were correlated with sugar content to further explore in three varieties by qRT-PCR. Based on the qRT-PCR result, *AaVINV2* and *AaVINV3* were found to be extremely highly expressed in QH, showing a strong correlation with glucose levels (Figure S5), which might contribute to sucrose accumulation in the fruits. However, five *AaNINVs* were highly expressed in KKW but expressed at low levels in the other varieties. These results suggest that the functions of *AaINVs* are diversified and complex. Studies have shown that *INVs* significantly effect sugar content in fruits [5,25]. Moreover, a subset of 18 to 25 genes exhibited distinct expression patterns across different kiwifruit varieties (Figure 6C,D), thereby suggesting a potential association with fruit functionality. The soluble sugars in kiwifruit, including glucose, fructose, sucrose and oligosaccharides, vary significantly among cultivars, providing a system to explore sugar accumulation mechanisms. Future research should focus on functional validation, including gene knockdown and overexpression studies, across a broader range of varieties to better elucidate the specific mechanisms by which *AaINV* family members regulate the complex processes of sugar metabolism and accumulation.

The modulation of sugar metabolism by the transcription factors (e.g., bHLH, ERF, NAC and MYB) has been confirmed in different horticulture crops [25,27,52]. Here, we primarily identified bHLHs and AP2/ERF-ERFs in kiwifruits, which were significantly different and highly correlated with sugar content (Figure 7 and Figure S6). As one of the largest TF families in plants, some members of the bHLH family are indicated as the key enzyme involved in carbohydrate accumulation, such as that of sorbitol, sucrose and starch in fruits [53]. ERFs are transcriptional regulators that mediate starch degradation, soluble sugar accumulation and ethylene-dependent gene expression in plants. These TFs are closely linked to fruit ripening [26,54,55]. To determine whether these TFs are involved in sucrose metabolism, we analyzed their correlations with the 42 *AaINV* genes. The results revealed significant correlations between the TFs and the expression of 14 *AaINV* genes (Figure 7). Taken together, these findings suggest that the ripening process of kiwifruit is governed by intricate regulatory networks.

In summary, a genome-wide identification and analysis of *AaINV* genes involved in sugar metabolism and accumulation were conducted in kiwifruit. A total of 102 *AaINV* genes were identified and categorized into three subgroups based on the phylogenetic tree, with the results further corroborated by gene structural domain and motif analysis. Additionally, differential expression patterns and regulatory networks offer valuable insights into the physiological functions of *INV* genes in kiwifruit.

## 4. Materials and Methods

### 4.1. Identification of INVs in A. arguta Genome

The genome details of *A. arguta* were downloaded from the China National GeneBank (Table S1). The candidate INV members were predicted using HMMER v3.3.2 [56] and PFAM [57] according to the *INV* conserved domain (NINV: PF12899; CWINV: PF08244 and PF00251; and VINV: PF11837, PF08244 and PF00251). Then, these members were further confirmed by a local BLASTP v2.15.0+ (E ≤ 1 × 10^−5^) search against 17 *INV* genes from *Arabidopsis* (Table S1). The redundant hits obtained from the Hidden Markov Model (HMM) profiles and BLASTP v2.15.0+ searches were manually eliminated. The physicochemical properties (e.g., AA, MW and theoretical pI) of AaINV proteins were calculated via ExPASy [58]. The subcellular localization of candidates was predicted by subCELlular LOcalization predictor: CELLO v2.5 (http://cello.life.nctu.edu.tw/ (accessed on 10 October 2024)).

### 4.2. Phylogenetic Tree Analysis

To classify the evolutionary relationships of the *INV* gene family, the INV protein sequences of *Arabidopsis*, *A. arguta*, *A. chinensis*, *A. eriantha*, *A. hemsleyana* and *A. rufa* were imported into MEGA v11 [59], and multiple sequence alignments were performed using MUSCLE with the default parameters. The phylogenetic tree based on the alignments was constructed using the neighbor-joining (NJ) method with Bootstrap tests on 1000 resamples.

### 4.3. Chromosomal Location, Gene Duplication and Collinearity Analyses

The physical position of *AaINV* genes on the chromosomes was obtained from the database of the *A. arguta* genome using the R package Circlize v0.4.15 [60]. The possible segmental duplication and tandem duplication events were analyzed through BLASTP (E ≤ 1 × 10^−5^) and MCScanX in TBtools v2.084 [61,62]. The JCVI was adopted to conduct the collinearity analysis of the orthologous *INV* genes between *A. arguta* and *Arabidopsis*, *A. chinensis* and *A. eriantha* [63]. To estimate the evolution of *AaINV* genes, KaKs_Calculator v2.0 was used to compute the nonsynonymous (*Ka*) and synonymous (*Ks*) substitution ratio of duplicate gene pairs, with *Ka*/*Ks* < 1 or *Ka*/*Ks* > 1 indicating purifying selection or positive selection, respectively [64].

### 4.4. Gene Structure, Conserved Motif and Domain Analysis of AaINV Members

The conserved structural domains of kiwifruit AaINV proteins were extracted from GSDS v2.0 [65]. The motifs of kiwifruit AaINV proteins were analyzed using the Multiple Em for Motif Elicitation (MEME) v5.5.3 with the motif number set to 10 [66]. In order to investigate the cis-acting regulatory elements, the 2k bp upstream of the coding regions of the candidates were retrieved from the kiwifruit genomes (*A. arguta* “M1”). The cis-acting elements were predicted by PlantCARE [67].

### 4.5. Expression Profiles Analysis of AaINVs in A. arguta Based on RNA-Seq Data

To analyze the *AaINV* genes expression profile at fruit-ripening stages, the raw reads of the nine mature fruit samples were generated from three *A. arguta* varieties, namely “Kukuwa” (KKW, full-green flesh), “Qinhuang” (QH, yellow) and “Xianziguang” (XZG, purple). RNA-seq quantitative analysis was completed through filtering adapters and low-quality reads via Trimmomatic v0.33 [68]. The obtained clean reads were then aligned to the reference genome of *A. arguta* “M” by HISAT2 v2.2.1 [69]. Subsequently, the expression levels of *AaINV* genes at different varieties were quantified using FPKM values, and the heatmaps of the expression level of these candidates were created by TBtools v2.084 [62]. The differentially expressed genes (DEGs) between different varieties were identified via DESeq2 based on the following criteria: log2Fold Change > 1 with adjusted *p*-values < 0.05 [70]. GO enrichment analysis was determined with clusterProfiler v4.0 [71]. Additionally, metabolome profiles were assessed by Metware Biotechnology Co., Ltd. (Wuhan, China) using widely targeted metabolome analysis [72]. Metabolite quantification and annotation were carried out using scheduled multiple reaction monitoring methods and the self-built MetWare database and other public databases, respectively. The Kruskal–Wallis test was used to compare any significant differences in metabolite levels. Statistical significance was considered at *p* < 0.05, and the values are presented as the mean ± SD from two independent samples. The sugar metabolic regulatory networks were constructed through combining the Pearson correlation coefficient (PCC > 0.9, *p* < 0.05) between TFs and structural genes with Cytoscape v3.7.0 [73].

### 4.6. Experimental Validation of AaINV Gene Expression Levels by qRT-PCR

To validate the RNA-seq results, we selected eight *AaINV* genes (*AaVINV2*, *AaVINV3*, *AaNINV3*, *AaNINV28*, *AaNINV35*, *AaNINV50*, *AaNINV62* and *AaNINV63*) that showed a correlation with sugar metabolites. The expression levels of these genes were further confirmed using qRT-PCR across three *A. arguta* varieties, with Actinidia β-actin serving as the internal control for normalization [74]. Total RNAs were isolated from KKW, QH and XZG fruits by an RNAprep Plant Kit (Tiangen, Beijing, China), and cDNA was synthesized as described by Jia et al. [33]. The qRT-PCR was carried out with SYBR Green PCR Master Mix (Takara, Kyoto, Japan) using a Bio-Rad Real-time PCR System. Each gene was repeated six times, and the data were processed using the 2^−ΔΔCT^ method.

## Data Availability

The data are contained within the Supplementary Materials.

## Supplementary Materials

The following supporting information can be downloaded at: https://www.mdpi.com/article/10.3390/ijms26052150/s1.
