# Supplementary material for: Genome-Wide Identification and Expression Profiling of the Invertase Genes Involved in Sugar Metabolism and Accumulation in Actinidia arguta
Source: Int J Mol Sci. 2025 Feb 27;26(5):2150. doi: 10.3390/ijms26052150 (PMC11899927; doi:10.3390/ijms26052150)
Supplement: Supplementary file 1 [file ijms-26-02150-s001.zip › ijms-3419610-supplementary/Supporting information/Supporting information.pdf]

Supporting information

Supporting Tables (eight Tables, Tables S1-S8)

Supporting Figures (six Figures, Figs. S1-S6)

Supporting Tables

Table S1 Genome information for genome-wide identification of *INV* genes in this study.

Table S2 Characteristics of 102 INV proteins in *A. arguta*.

Table S3 The number of *INV* genes in *Arabidopsis* and *Actinidia* used in this study.

Table S4 The three duplicated types of *AaINV* genes in kiwifruit genome.

Table S5 Homologous gene pairs and Ka/Ks values in *A. arguta*.

Table S6 Overview of the Illumina HiSeq in the three kiwifruit varieties.

Table S7 Representative metabolites involved in sugar metabolism in the three kiwifruit varieties.

Table S8 Pearson correlation coefficient between the 42 *AaINVs*, 14 metabolites and TFs.

Supporting Figures

Figure S1 Subcellular localization map of AaINVs. The different colors and shapes were used to recognize various types.

Figure S2 Correlation analysis of *AaINV* genes in kiwifruit. Blue and red colors represent positive and negative correlations with significant p-values (p-value<0.05), respectively.

Figure S3 Heatmap of *AaINV* genes expression from the three kiwifruit varieties.

Figure S4 GO enrichment analysis of the 102 *AaINV* members in kiwifruit.

Figure S5 Pearson correlation coefficients (PCC) between *AaINVs* expression and sugar concentrations in kiwifruit. Red and blue color notes positive and negative correlations with gene expression, respectively.

Figure S6 Heatmaps of differentially expressed TFs among the three kiwifruit.

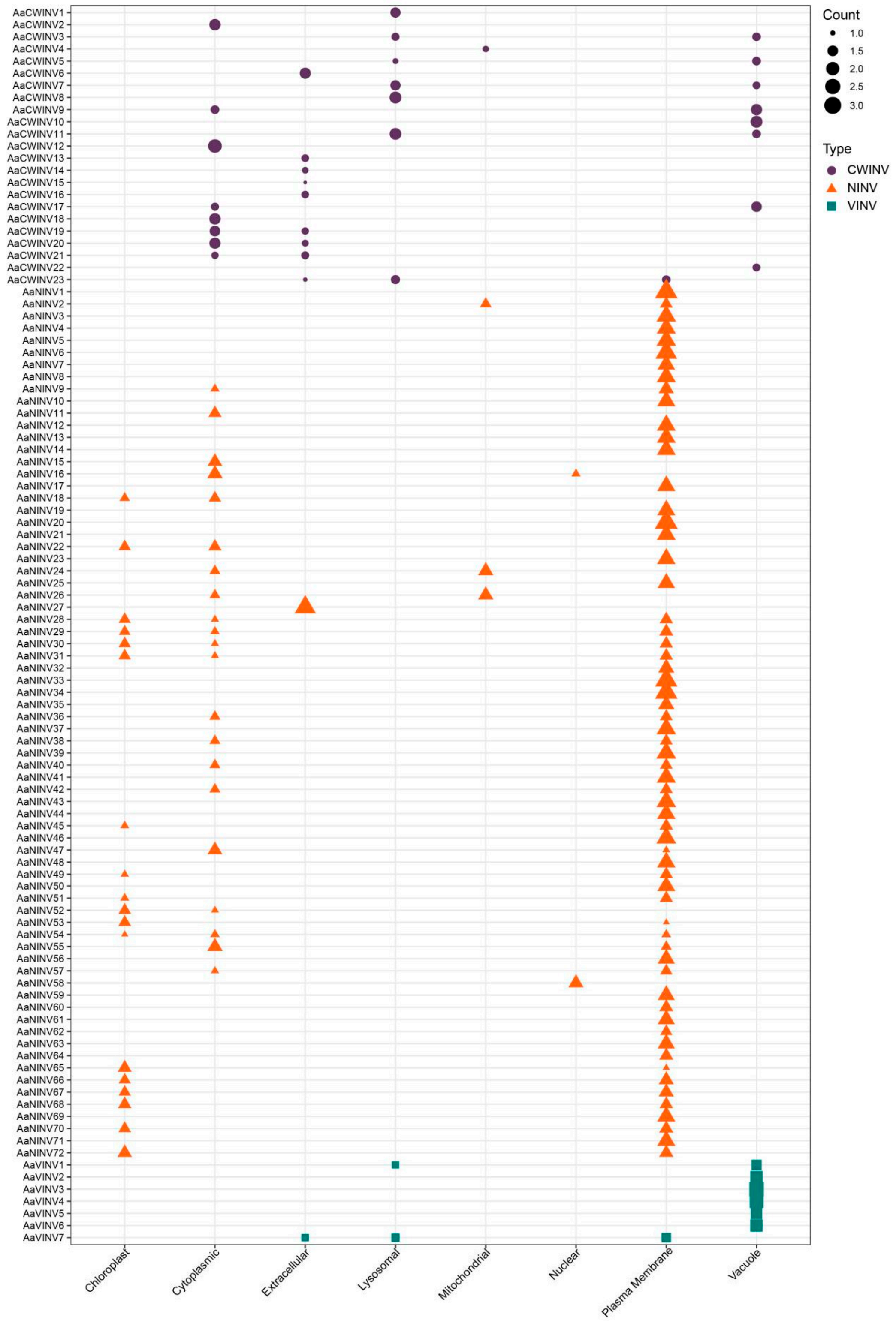

Figure S1 Subcellular localization map of *AaINV*s. The different colors and shapes were used to recognize various types.

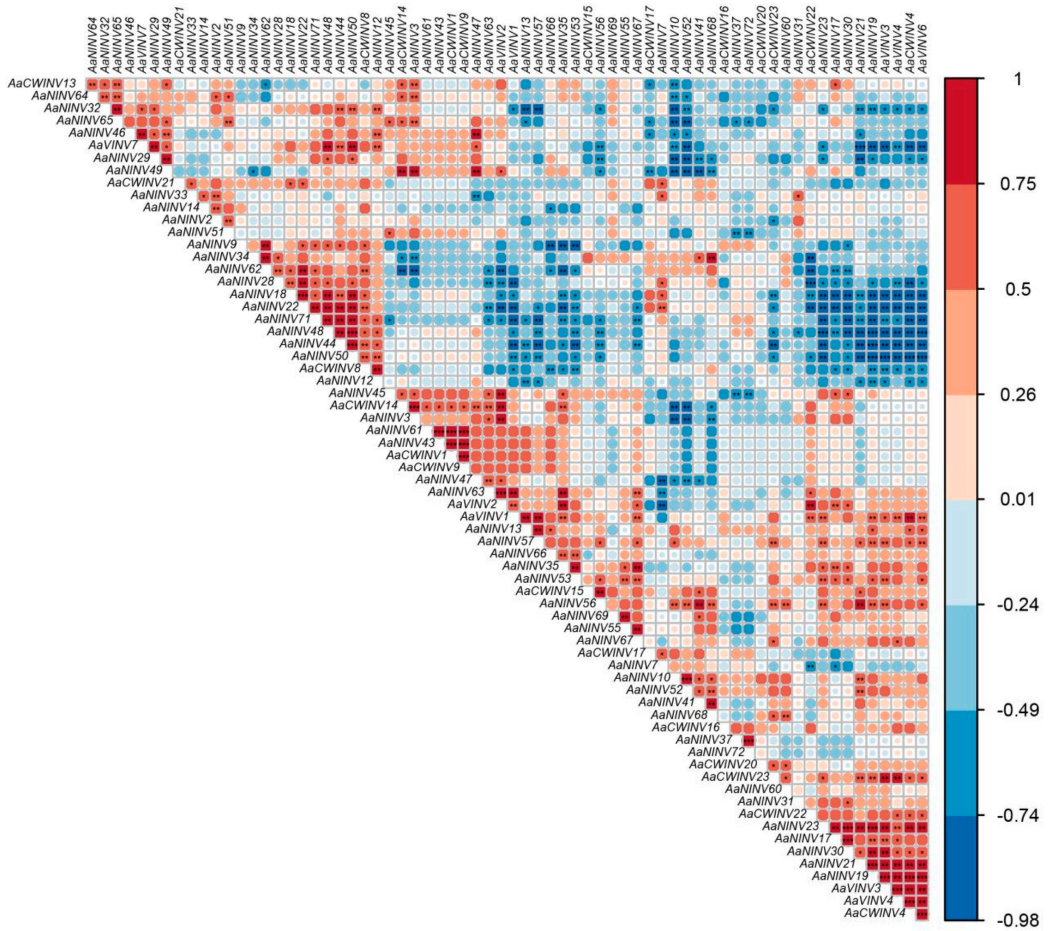

Figure S2 Correlation analysis of *AaINV* genes in kiwifruit. Blue and red colors represent positive and negative correlations with significant p-values (p-value<0.05), respectively.

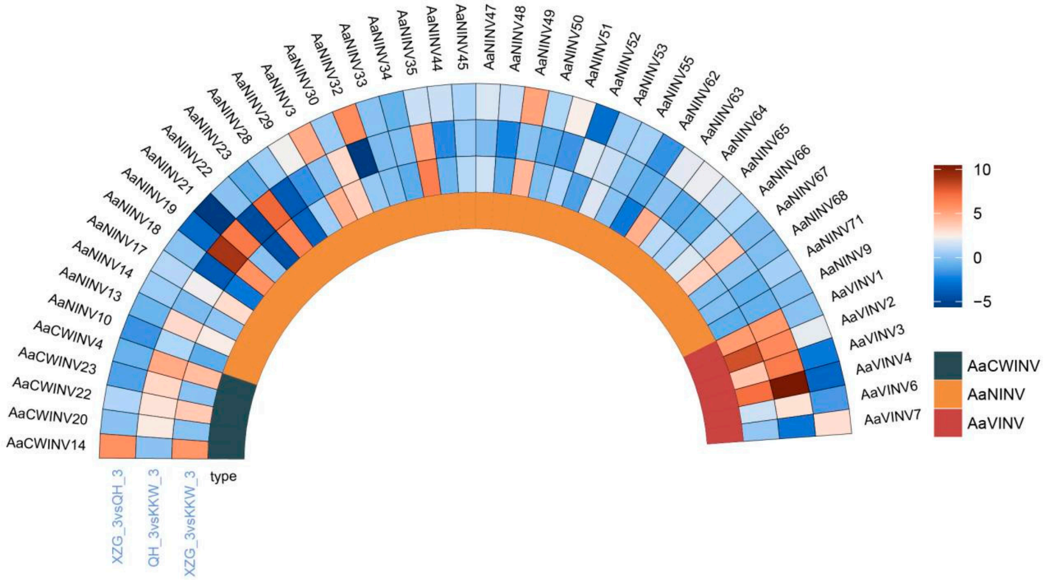

Figure S3 Heatmap of *AaINV* genes expression from three kiwifruit varieties.

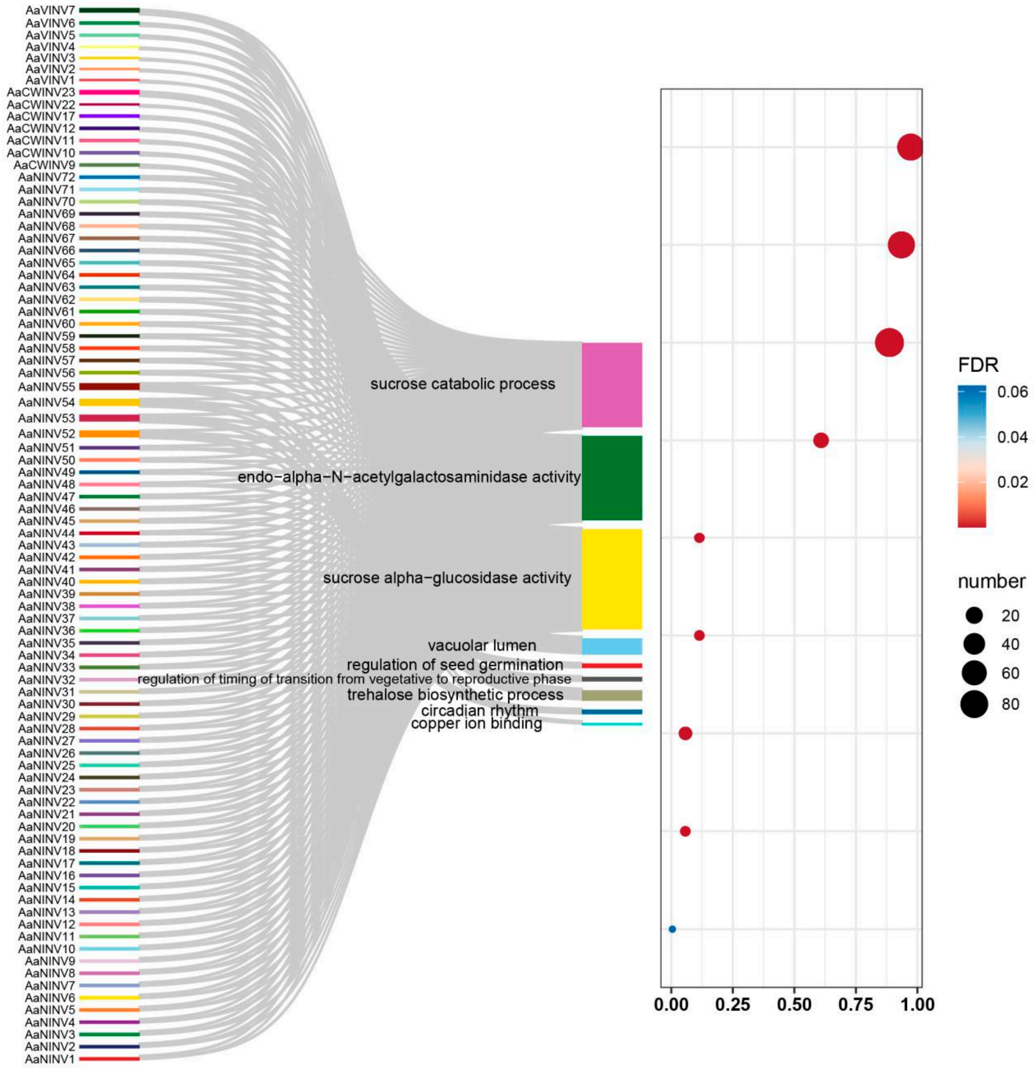

Figure S4 GO enrichment analysis of the 102 *AaINV* members in kiwifruit.

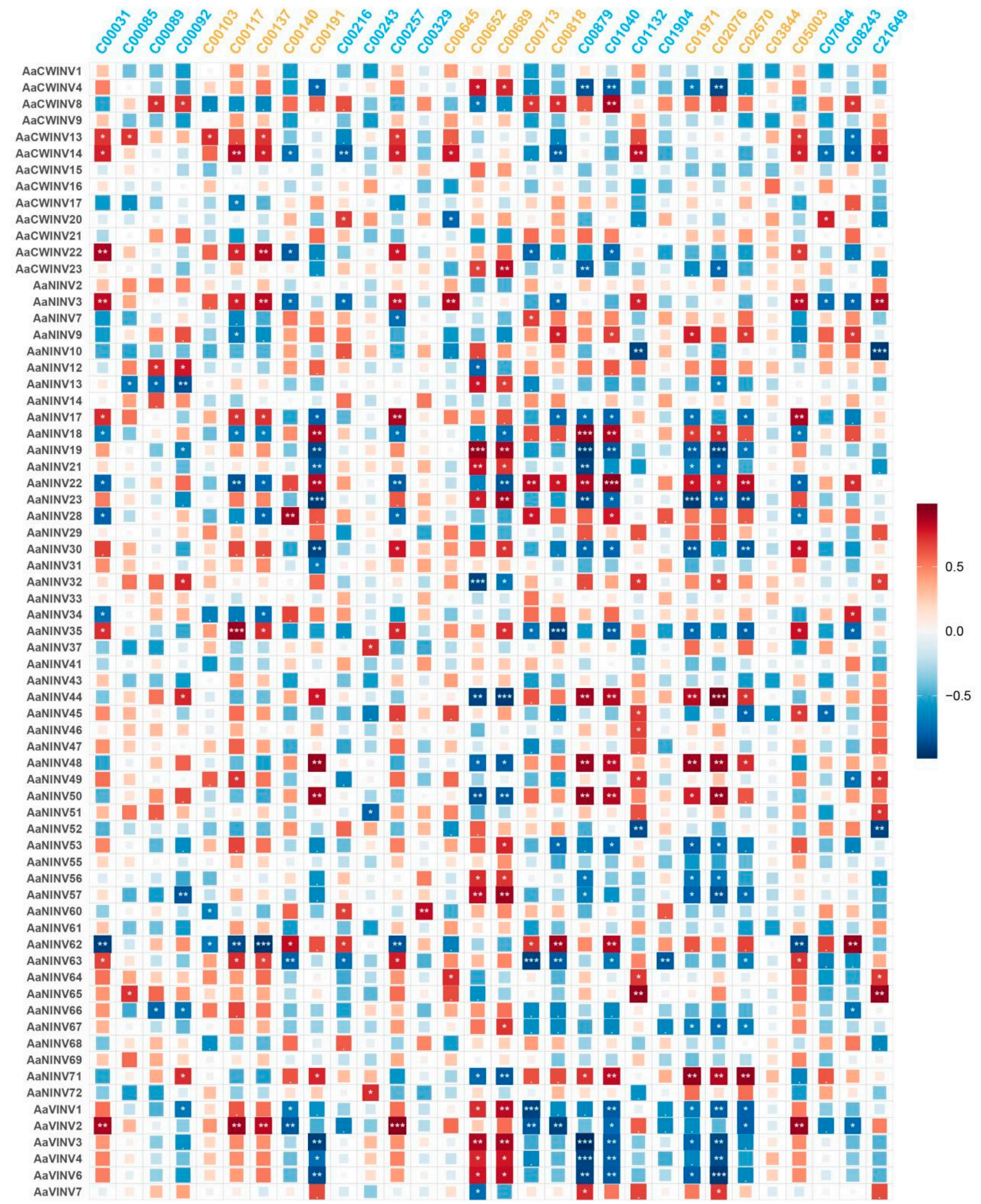

Figure S5 Pearson correlation coefficients (PCC) between *AaINVs* expression and sugar concentrations in kiwifruit. Red and blue color notes positive and negative correlations with gene expression, respectively.

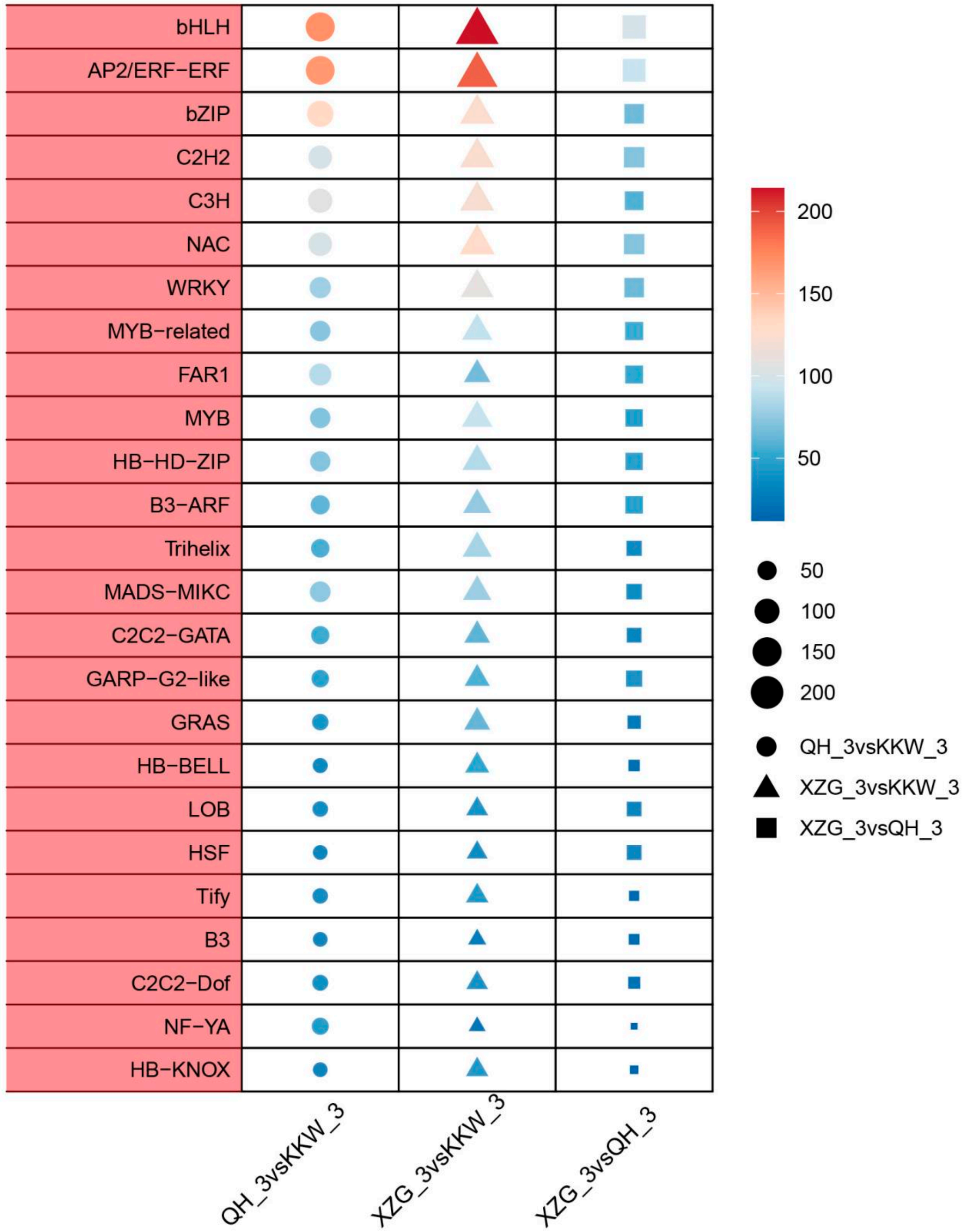

Figure S6 Heatmaps of differentially expressed TFs among the three kiwifruit.
